# Frailty status and chronic lung disease: a contextual and methodological discussion

**DOI:** 10.1007/s40520-025-02943-7

**Published:** 2025-02-27

**Authors:** Gui-Yu Feng, Guo-Sheng Li, Tao Huang, Hua-Fu Zhou

**Affiliations:** 1 Department of Cardiothoracic Surgery, The First Affiliated Hospital of Guangxi Medical University, Guangxi Zhuang Autonomous Region, No. 6, Shuangyong Road, Nanning, 530021 P. R. China; 2 Department of Cardiothoracic Vascular Surgery, The Affiliated Hospital of Youjiang Medical University for Nationalities, Baise, P. R. China; 3 Key Laboratory of Metabolic Diseases of Baise, The Affiliated Hospital of Youjiang Medical University for Nationalities, Baise, P. R. China

We appreciate the attention that Fan et al. devoted to our study [1] and the insightful comments they provided. Below, we offer our responses to the issues raised by these authors.

Fan et al. stated that younger frail individuals (< 65 years) showed a higher risk of chronic lung disease (CLD) (hazard ratio [HR] = 1.87) compared to older adults. However, this interpretation may misrepresent the results presented in Table 3 of our previous study [1]. The table includes findings from a stratified analysis aimed at exploring the risk of CLD associated with frailty status. In this table, the comparison groups are frail and pre-frail individuals, with robust individuals (not older adults or any other group) serving as the reference group. The HR of 1.87 indicates that, among individuals aged under 65 years, those who are frail have 1.87 times the risk of developing CLD compared with robust individuals. We acknowledge that explicitly indicating the reference group in the original text could have clarified this point for readers. Nonetheless, the current context and the results shown in Table 3 clearly support our conclusion [1].

Regarding confounding factors, Fan et al. suggested that we did not account for physical activity, hypertension, or diabetes. However, as detailed in Supplementary Material 1 in our previous study [1], these factors were already included in the calculation of frailty index (FI) scores. The FI is a comprehensive assessment index [2]; in our study, it included not only physical activity (e.g., “difficulty climbing several flights of stairs without resting”), hypertension (“high blood pressure”), and diabetes but also psychological, cognitive, and laboratory measures (e.g., “mean peak expiratory flow reading”) [1]. Given this context, we submit that physical activity, hypertension, and diabetes are components of FI and should not be included as separate covariates. If this were done, it might lead to an overestimation of their effects on CLD.

We agree with Fan et al.’s suggestion that restricted cubic spline (RCS) analysis could potentially capture nonlinear relationships. However, it is important to note that, when a linear relationship is evident, the advantages of RCS may not provide additional value; indeed, such analysis could increase the model’s complexity unnecessarily. In our study, a linear relationship was established via Cox analysis using frailty status as a categorical variable (robust, pre-frail, and frail). Sensitivity analyses, including the accelerated failure time model and Cox analysis with FI as a continuous variable, further confirmed this linear relationship. In addition, findings across both cohorts (CHARLS and ELSA) indicated that both frail and pre-frail individuals had a significantly higher risk of CLD compared with robust individuals. Ultimately, these results do not suggest a potential nonlinear relationship between FI status and CLD risk. Moreover, the established linear relationship provides a straightforward and effective method for assessing the association between the two. If future studies reveal a potential nonlinear relationship, the application of RCS analysis may provide additional insights into this topic.

## Data Availability

No datasets were generated or analysed during the current study.

## References

[1] Feng GY, Li JX, Li GS, Liu J, Gao X, Yan GQ et al (2024) Association between frailty status and risk of chronic lung disease: an analysis based on two national prospective cohorts. Aging Clin Exp Res 36:215 39520636 10.1007/s40520-024-02867-8 PMC11550224

[2] Mitnitski AB, Mogilner AJ, Rockwood K (2001) Accumulation of deficits as a proxy measure of aging. ScientificWorldJournal 1:323–336 12806071 10.1100/tsw.2001.58 PMC6084020

